# The gut–brain axis in appetite, satiety, food intake, and eating behavior: Insights from animal models and human studies

**DOI:** 10.1002/prp2.70027

**Published:** 2024-10-17

**Authors:** Georgia S. Clarke, Amanda J. Page, Sally Eldeghaidy

**Affiliations:** ^1^ School of Biomedicine The University of Adelaide Adelaide South Australia Australia; ^2^ Robinson Research Institute The University of Adelaide Adelaide South Australia Australia; ^3^ Nutrition, Diabetes and Gut Health, Lifelong Health Theme South Australian Health and Medical Research Institute, SAHMRI Adelaide South Australia Australia; ^4^ Division of Food, Nutrition and Dietetics School of Biosciences, University of Nottingham Nottingham UK; ^5^ Sir Peter Mansfield Imaging Centre School of Physics and Astronomy, University of Nottingham Nottingham UK

**Keywords:** gut–brain axis, hedonic, homesostatic, obesity, GLP‐1, brain imaging, fMRI

## Abstract

The gut–brain axis plays a pivotal role in the finely tuned orchestration of food intake, where both homeostatic and hedonic processes collaboratively control our dietary decisions. This interplay involves the transmission of mechanical and chemical signals from the gastrointestinal tract to the appetite centers in the brain, conveying information on meal arrival, quantity, and chemical composition. These signals are processed in the brain eventually leading to the sensation of satiety and the termination of a meal. However, the regulation of food intake and appetite extends beyond the realms of pure physiological need. Hedonic mechanisms, including sensory perception (i.e., through sight, smell, and taste), habitual behaviors, and psychological factors, exert profound influences on food intake. Drawing from studies in animal models and human research, this comprehensive review summarizes the physiological mechanisms that underlie the gut–brain axis and its interplay with the reward network in the regulation of appetite and satiety. The recent advancements in neuroimaging techniques, with a focus on human studies that enable investigation of the neural mechanisms underpinning appetite regulation are discussed. Furthermore, this review explores therapeutic/pharmacological strategies that hold the potential for controlling food intake.

## Graphical abstract

Homeostatic and hedonic interactions in reward, motivation and food intake.

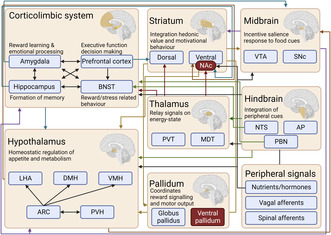

**Abbreviations**
AgRP: agouti‐related peptide
ARC: arcuate nucleus
BNST: bed nucleus of the stria terminalis
BOLD: blood oxygenated level dependent
CART: cocaine and amphetamine‐regulated transcript
CCK: cholecystokinin
DMN: dorsomedial nucleus
EEC: enteroendocrine cells
fMRI: functional magnetic resonance imaging
GABA: γ‐aminobutyric acid
GIP: glucose‐inhibitory polypeptide
GLP‐1: glucagon‐like peptide 1
GLP‐1R: GLP‐1 receptor
LHA: lateral hypothalamic area
MRI: magnetic resonance imaging
MC4R: melanocortin 4 receptor
α‐MSH: alpha‐melanocyte stimulating hormone
NAc: nucleus accumbens
NTS: nucleus of the solitary tract
PYY: peptide YY
NPY: neuropeptide Y
OFC: Orbitofrontal cortex
PBN: parabrachial nucleus
PVN: paraventricular nucleus
PVT: paraventricular thalamus
PFC: prefrontal cortex
POMC: pro‐opiomelanocortin
SNc: substantia nigra pars compacta
VP: ventral pallidum
VS: ventral striatum
VTA: ventral tegmental area
VMN: ventromedial hypothalamic nucleus
Y2R: PYY receptor

## 1 INTRODUCTION

The interplay between the gastrointestinal tract and the brain, the gut–brain axis, emerges as a central coordinator of our daily dietary choices. Food intake is a carefully regulated behavior existing as a cycle between hunger, satiation, and satiety. Hunger refers to the state prompting food intake, while satiation is the feeling of fullness and controls meal size (meal duration) and satiety is the postprandial events which terminate a meal and determines the time before hunger is perceived again (inter‐meal duration).^1^ It is well established that the regulation of this cycle extends beyond homeostatic control, whereby energy requirements are matched to energy demands, and involves hedonic processes including sociocultural aspects and environmental factors. Sensory perception, including sight, smell, taste, and palatability, can often override homeostatic control.

Much of our understanding of the homeostatic and hedonic processing of food intake has been derived from animal studies. Translating these cellular mechanisms observed in animal models to humans is complex and not straightforward. However, recent advances in non‐invasive neuroimaging techniques including anatomical, functional, or metabolic imaging, have enabled the assessment of food intake and its dysregulation, providing valuable insights into human food intake regulation.^2^, ^3^ For example, structural magnetic resonance imaging (MRI) techniques allow the measurement of the neuroanatomical properties of the brain, including global and regional gray matter and white matter volumes and microstructure. These techniques have been used to compare subjects with altered eating behavior to healthy controls. Evidence from a voxel‐based gray matter meta‐analysis,^4^ across 14 structural MRI studies, reported a decrease in gray matter volume in reward network and sensorimotor processing areas in overweight or obese individuals, suggesting an underlying structural basis for reward processing and sensorimotor processing dysregulation in overweight and obese subjects. While structural MRI allows us to investigate the anatomical and morphological characteristics underpinning reward and satiety processing, functional MRI (fMRI) offers insights into the dynamic aspects of brain activity during food‐related tasks. In typical food‐related experiments, brain activity patterns are measured through hemodynamic changes, typically using blood oxygenation level dependent (BOLD) technique or BOLD contrast, which are associated with neural activation in response to food regulation and responses. Various fMRI designs are employed to investigate food intake, aiming to investigate the neural mechanisms underlying food reward, satiety, and responses to food cues such as images or smells of food. In these studies, participants are often exposed to pictures of food while their brain activity is recorded using fMRI. When a brain region is more active, it receives more blood flow, and this change can be detected and visualized by the fMRI. Additionally, the statistical relationship or synchronization between neural activities in different regions, either during task or at rest, can be measured using functional connectivity techniques. Resting‐state fMRI examines brain activity when a person is not engaged in any specific task, allowing for a broad, network‐based approach to studying the brain's functional architecture. This is typically used to assess alterations in functional connectivity between fed and fasted states. In contrast, task‐based connectivity studies aim to understand the dynamic changes in connectivity as the brain engages in a specific task.

This comprehensive review aims to explore our current understanding of food intake regulation drawing from studies in animal models and human fMRI studies. It will cover both central and peripheral mechanisms involved in the regulation of food intake. The review will also discuss promising therapeutic/pharmacological strategies that could be effective in the control of food intake.

## 2 FOOD INTAKE REGULATION

### 2.1 Central mechanisms

#### 2.1.1 Homeostatic

**FIGURE 1:**
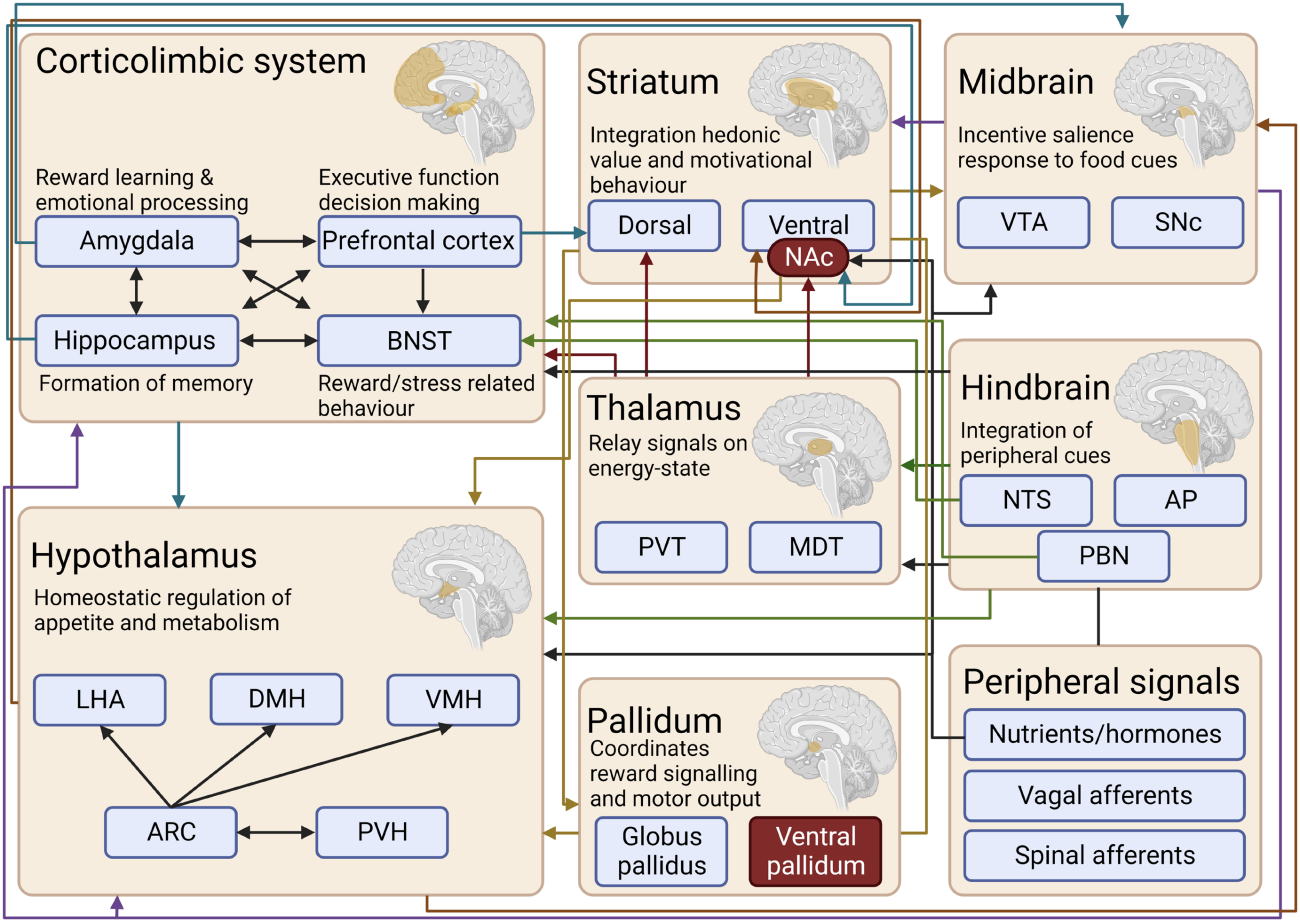
Schematic of the central neural circuitry in reward, motivation, and food intake. Peripheral neural and hormonal signals are integrated in the hindbrain and hypothalamus. These signals are relayed through the thalamus and midbrain and integrated with corticolimbic signals in the striatum. Projections from the striatum to the pallidum and hypothalamus enable coordination of motivated behavioral responses and reward seeking behavior. Hedonic “hotspots” are shown in red. AP, area postrema; ARC, arcuate nucleus; BNST, bed nucleus of the stria terminalis; DMH, dorsal medial hypothalamus, LHA, lateral hypothalamic area; MDT, mediodorsal thalamic nucleus; NAc, nucleus accumbens; NTS, nucleus of the solitary tract; PBN, parabrachial nucleus; PVH, paraventricular hypothalamus; PVT, paraventricular thalamic nucleus; SNc, substantia nigra pars compacta; VMH, ventromedial hypothalamus; VTA, ventral tegmental area. Created with BioRender.com.

The hypothalamus (Figure 1) is the central region in the homeostatic control of food intake regulation and involves complex communication between the arcuate nucleus (ARC), paraventricular nucleus (PVN), lateral hypothalamic area (LHA), ventromedial hypothalamic nucleus (VMN), and dorsomedial nucleus (DMN).^5^ The ARC, situated in the medial basal hypothalamus, is a key region in this network. Its strategic location, near the fenestrated capillaries of the median, allows it to effectively detect hormonal and nutrient signals from the periphery, as well as signals from the nucleus of the solitary tract (NTS).^5^

Within the ARC, there are two neuronal populations which regulate appetite, the pro‐opiomelanocortin (POMC)/cocaine and amphetamine‐regulated transcript (CART)‐expressing neurons and neuropeptide Y (NPY)/agouti‐related peptide (AgRP)‐expressing neurons. These neuronal subtypes also make extensive connections with other neuronal populations within the brain although their functions oppose, such that POMC/CART neurons are anorexigenic and NPY/AgRP‐expressing neurons are orexigenic.^5^ In POMC neurons, the POMC gene encodes for a precursor polypeptide, that once cleaved, yields many neuropeptides such as alpha‐melanocyte stimulating hormone (α‐MSH).^6^ α‐MSH is a key hormone to suppress appetite, with effects on food intake well documented (refer to review^6^, ^7^). Briefly, when α‐MSH is administered centrally, it leads to reduced food intake in rats, mice, and goldfish (as reviewed^7^). These actions of α‐MSH are mediated through actions on the melanocortin 4 receptor (MC4R) which is expressed highest in the PVN, although they can be found in other brain regions (as reviewed^5^). Furthermore, mice that lack α‐MSH and POMC exhibit hyperphagia and obesity,^7^ with the hyperphagia reversed by α‐MSH administration (reviewed by^5^). In contrast, the NPY/AgRP neurons are activated by fasting and inhibited by feeding.^6^ The feeding response of NPY is mediated via NPY receptors (Y receptors), specifically Y1 and Y5 receptors located in the ARC, PVN, DMN, and other regions, including the LHA.^8^ Furthermore, AgRP suppresses food intake by acting as an antagonist at MC4R.^5^ NPY/AgRP neurons also inhibit POMC neurons by secreting inhibitory γ‐aminobutyric acid (GABA).^5^

Until recently, most of our understanding of the hypothalamus' role in regulating food intake came from animal studies, primarily due to the technical challenges of imaging this brain region with fMRI. The small structure and deep location of the hypothalamus make it difficult to image in human subjects. In addition, its proximity to air/tissue boundaries leads to distortion in the signal and susceptibility to artifacts caused by physiological factors, such as pulse and respiration.^9^ Despite these challenges, advancements in scanning technology have now made it feasible to image the hypothalamus. This has led to a series of human fMRI studies aimed at evaluating the hypothalamic response to various food‐related stimuli, under conditions of both satiety and hunger. These studies frequently compare the brain responses between individuals with normal eating patterns and those of altered eating behaviors. For example, several studies have found that administering glucose to healthy participants leads to a dose‐dependent reduction in the hypothalamic BOLD signal within the PVN and the VMH, both of which have an anorexigenic role.^10^, ^11^ This reduction in BOLD signal is more pronounced in patients with anorexia nervosa compared to healthy controls, suggesting potential neural mechanisms that underlie altered eating behaviors in these individuals.^12^ Interestingly, oral glucose intake triggers two peaks of hypothalamic BOLD response in healthy subjects: an initial response right after ingestion, followed by another peak around 10 min later.^13^ This delayed response is associated with a negative response in the medial hypothalamus, correlating with fasting plasma insulin levels,^13^ reflecting the role of the hypothalamus‐modulating insulin secretion for glucose regulation. More recently, the use of high‐resolution fMRI imaging was applied to the hypothalamus to investigate glucose metabolism based on individual hypothalamic nuclei. These studies reported decreased activity in the VMH and increased activity in the LHA between 10‐ and 40‐min post‐glucose ingestion.^14^ Furthermore, a decrease in activity in the arcuate nucleus was observed, followed by a rise in blood insulin during the first 10 min after glucose ingestion,^14^ further highlighting the complex role of the hypothalamus in energy balance and metabolism.

#### 2.1.2 Hedonic system

In addition to homeostatic signals, food intake is impacted by food cues, social factors, and memory, promoting consumption of palatable food^15^ beyond homeostatic need and coordinated by the hedonic system. The hedonic system can be split into “liking” and “wanting” components, coordinated by distinct but interconnecting circuits.^16^, ^17^ These reward circuits are complex with many regions projecting to and receiving input from areas involved in the homeostatic control of food intake, including the hypothalamus and hindbrain (Figure 1).

The corticolimbic system is involved in the executive, emotional, and mnemonic processing of food intake.^18^ It receives input from the paraventricular thalamus (PVT), midbrain, hypothalamus, and parabrachial nucleus (PBN) and sends projections to the striatum and hypothalamus^18^ (Figure 1). The striatum is an integration site, with the dorsal striatum integrating inputs from the prefrontal cortex (PFC), motor cortex, thalamus, and the midbrain substantia nigra pars compacta (SNc) region and sending projections to the globus pallidus of the basal ganglia^19^ and the nucleus accumbens (NAc) of the ventral striatum (VS) integrating signals from the PFC, hippocampus, amygdala, PVT, midbrain ventral tegmental area (VTA) and hypothalamus and sending projections to the ventral pallidum (VP), hypothalamus and VTA.^20^, ^21^, ^22^ These circuits determine the hedonic value of food with, for example, “hedonic hotspots” in NAc and VP contributing to the “liking” of foods.^23^, ^24^, ^25^, ^26^ The mesolimbic system is important for encoding the “wanting” of food as well as conditioned responses to food cues.^27^, ^28^, ^29^, ^30^, ^31^ This system connects the midbrain, specifically the VTA and substantia nigra pars compacta (SNc), with the striatum, corticolimbic system, and hypothalamus.^32^, ^33^

Functional MRI studies have revolutionized our understanding of the complexities of reward and hedonic processing, and have shown increased activation in hedonic pathways, including the VTA, NAc, and orbitofrontal cortex (OFC), when individuals are exposed to food cues, especially those high in sugar^34^ or fat^35^ content. A study by Gearhardt et al. demonstrated how the brain's reward centers are activated in response to food cues, similar to responses observed in addictive behaviors.^36^ When examining brain regions activated by cravings, the insula, involved in sensory processing, and the prefrontal cortex, linked to decision making and impulse control, were activated.^37^ These findings suggest that food cravings might be linked to an intricate network involving sensory perception, memory, and reward anticipation. Understanding how the brain balances the reward of eating with signals of satiation is crucial in addressing issues like overeating and obesity.

Although the hypothalamus is involved in the homeostatic control of food intake and energy expenditure, it is also a key region in the hedonic circuitry. For instance, the LHA integrates homeostatic and hedonic signals to synchronize motivated and reward‐seeking behavior.^38^, ^39^ The LHA receives input from the PFC, basolateral amygdala, NAc and bed nucleus of the stria terminalis (BNST)^40^ and sends projections to the central amygdala, VTA, PVT lateral habenula,^41^, ^42^ supporting a role for the LHA as a reward center. The ARC is also implicated in the integration of homeostatic and hedonic cues, with POMC neurons projecting to the mesolimbic system, specifically the VTA and NAc.^43^, ^44^

In human subjects, resting‐state fMRI studies have revealed a functional link between the hypothalamus and brain regions associated with appetite.^45^ These studies demonstrated an increase in functional connectivity between the hypothalamus and the medial PFC^46^ and the insula cortex,^47^ during fasting in individuals with normal weight. Conversely, a decrease in hypothalamic functional connectivity was reported in the sated state compared to the fasted state.^48^ Interestingly, this decrease in hypothalamus connectivity at the sated state was less pronounced in overweight or obese individuals.^47^ Similar trends were observed in task‐based connectivity studies, where hypothalamic activity was shown to be modulated by both internal factors (such as hunger state) and external cues (i.e., food cues with varying caloric content) in individuals with and without obesity.^49^ However, participants of normal weight exhibited higher functional connectivity between the hypothalamus and dorsolateral PFC compared to those who were overweight or obese. This difference could potentially contribute to overeating in individuals with obesity.^49^

The central homeostatic and hedonic centers receive neural and/or hormonal input from the periphery, such as from the gastrointestinal tract and adipose tissue, although compared to the homeostatic system the mechanisms by which peripheral signals impact the hedonic system are less well defined.

### 2.2 Peripheral signals

#### 2.2.1 Gastrointestinal signals

The vagus nerve provides a two‐way communication between the gut and brain. Vagal afferents detect mechanical and chemical stimuli evoked within the gut in response to food intake. These signals are transmitted to the brainstem, specifically the NTS, where they are processed leading to either a vago‐vagal reflex, with vagal efferent signaling back to the GI tract impacting gut motility and enzyme secretion, or the relay of information to different regions of the brain involved in physiological and behavioral responses.^50^, ^51^, ^52^, ^53^, ^54^

The gastrointestinal tract orchestrates multiple signals which work in unison to terminate a meal at the appropriate time. For example, in human's nutrient ingestion induces an initial drop in intragastric pressure, induced via the vago‐vagal reflex control of gastric accommodation, followed by a gradual recovery.^55^ As discussed below, tension‐sensitive mechanosensitive afferents mediate gastric filling‐related satiety signaling,^56^ hence, relaxation of the stomach at the initiation of food intake will decrease activation of tension‐sensitive vagal afferents allowing the initial consumption of food. However, as feeding progresses and the stomach fills, intragastric pressure increases with a subsequent increase in tension‐sensitive vagal afferent satiety signaling and the termination of a meal. The importance of gastric accommodation in food intake regulation is highlighted in barostat studies, where impaired gastric accommodation is associated with early satiety and weight loss in patients with functional dyspepsia,^57^, ^58^ likely compounded by an increase in gastric tension‐sensitive vagal afferent mechanosensitivity.^59^ Therefore, it is crucial to have a clear understanding of the vagal afferent subtypes responsible for the initial gastric accommodation and the subsequent satiety signaling, as well as the different central pathways involved. This will enable more targeted treatment for diseases associated with food intake, such as obesity and functional dyspepsia.

Making up the gastrointestinal tract, the stomach and intestine are densely innervated by sensory vagal afferents which detect the quantity and chemical composition of a meal.^60^ There are several subtypes of vagal afferent based on the location of the sensory endings and the type of stimuli they respond to, including both mechanical and/or chemical signals. These vagal afferents then project centrally to the NTS, with cell bodies located in the nodose ganglia, where information is then integrated into the brainstem, limbic system, and hypothalamus to regulate food intake.^50^

#### 2.2.2 Volume‐dependent signals

One of the first satiation signals after food consumption arise from the increase in gastric volume as food fills the stomach, activating mechanosensitive gastric vagal afferents. Within the stomach, mechanosensitive vagal afferents can be separated into mucosal and tension‐sensitive afferents. Mucosal vagal afferents innervate the mucosal layer^60^ and respond to fine tactile stroking,^61^ which simulates food particles moving over the luminal surface. Although the function of these afferents is not clear, within the stomach, detection of food particle size is thought to regulate gastric emptying^62^; however, research is needed to confirm this in vivo. In comparison, tension‐sensitive vagal afferents respond to the stretching of the gastric wall,^61^ with sensory endings forming intraganglionic laminar endings within the myenteric plexus^63^ and intramuscular arrays in the circular and longitudinal muscle layers.^64^ Gastric balloon inflation models satiety caused by gastric distention and in humans, inflation of the proximal stomach with a bag induced the feeling of fullness.^65^ Furthermore, when a gastric balloon is filled with 400–800 mL of water, there is a volume dependent decrease in food intake.^66^ Similar findings can be replicated in rats, where food intake was reduced for up to 240 min compared to control rats (balloon remained deflated).^67^ Severing the vagal nerve of rats also increases food intake for 48 h, suggesting the inability to sense gastric volumes or the nutrient value of food.^67^ These results imply that pre‐absorptive satiety caused by stomach distension is the dominant signal generated by the stomach to reduce food intake. Indeed, two bariatric FDA‐approved bariatric procedures for obesity management currently target the stomach, namely intragastric balloons and endoscopic sleeve gastroplasty which impact tension‐sensitive vagal afferent responses through an increase in gastric content or a reduction in gastric volume respectively. In a randomized controlled trial placement of an intragastric balloon delayed gastric emptying and increased satiety,^68^ with the lifestyle intervention plus balloon reducing weight by 10.2% compared to 3.3% for the lifestyle intervention alone.^68^ Further, in a randomized controlled trial in patients with obesity those randomized to endoscopic sleeve gastroplasty and lifestyle modifications achieved 13.6% weight loss compared to 0.8% with lifestyle modifications alone^69^ demonstrating the potential of targeting gastric afferents in weight loss management. There are also tension‐sensitive vagal afferents in the small intestine and mechanical distension, via an intra‐intestinal saline infusion, inhibited subsequent food intake similar to intra‐gastric methods in mice.^60^

#### 2.2.3 Meal‐related enteroendocrine responses

##### Intestinal

**FIGURE 2:**
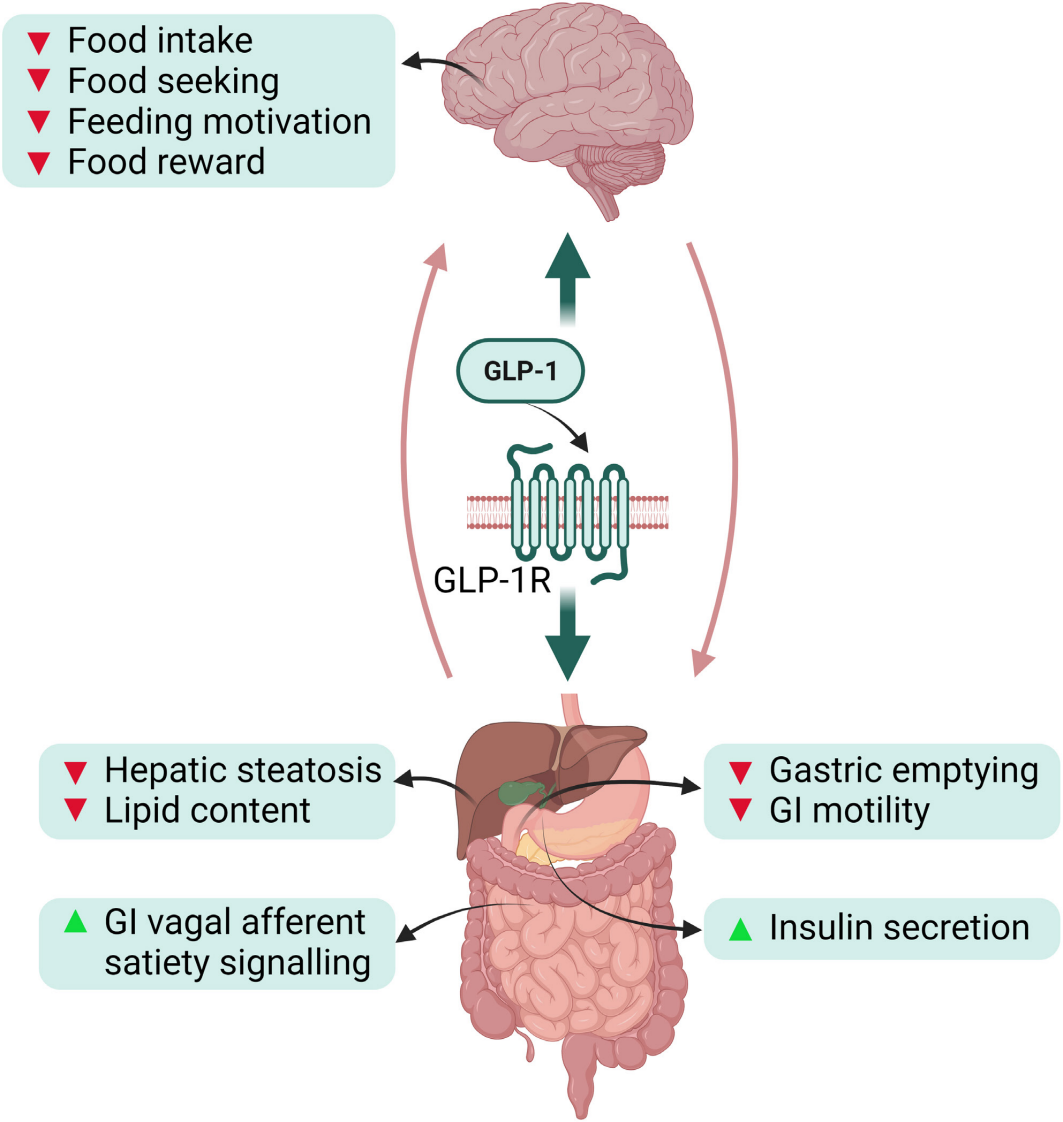
Potential mechanisms of weight loss caused by the effects of GLP‐1 and its analogues on the 7 transmembrane domain GLP‐1R along the gut–brain axis. The red arrowhead indicates a decrease and the green arrowhead indicates an increase. GI, gastrointestinal; GLP‐1, glucagon‐like peptide 1; GLP‐1R, glucagon‐like peptide 1 receptor. Created with BioRender.com.

Upon gastric emptying, macronutrients including proteins, carbohydrates, and lipids are broken down into smaller amino acids, monosaccharides, and fatty acids by digestive enzymes, which are then detected by chemoreceptors expressed on enteroendocrine cells (EECs).^70^ EECs that express chemoreceptors also express gut hormones, including cholecystokinin (CCK), glucagon‐like peptide 1 (GLP‐1; Figure 2) and peptide YY (PYY), and nutrient binding to these chemoreceptors culminates in hormone release.^71^ The secretion of these hormones exhibits both regional and nutrient specific patterns along the small intestine. CCK is secreted by I‐cells, which are localized in the proximal small intestine (i.e., duodenum, jejunum), and respond to fatty acids and protein by‐products.^72^ In comparison, GLP‐1 and PYY are secreted from L‐cells in response to all fatty acids, amino acids and proteins, with localization of L‐cells increasing down the cephalocaudal axis.^72^ CCK, GLP‐1, or PYY can have autocrine or paracrine effects by either directly activating or modulating vagal afferents^73^ or acting hormonally on peripheral and central tissues once in the circulation. For instance, CCK1 receptors (CCK1‐R) are located in the hypothalamus and hindbrain, with hypothalamic administration of CCK decreasing food intake.^74^ The ability of these hormones to reduce food intake is complex and has been reviewed in detail previously in a variety of animal models, including humans and rodents.^72^, ^75^, ^76^ Due to the short plasma half‐life, especially for active GLP‐1, actions on vagal afferent are more likely to mediate the effects of these gut hormones on food intake,^77^ especially considering vagal afferents express receptors for CCK (CCK1‐R,^78^, ^79^), PYY (Y2R,^80^), and GLP‐1 (GLP‐1R,^81^; Figure 2). The effect of these hormones on vagal afferents have been reported through multiple experimental rat studies, including PYY^82^ and CCK^83^ increasing intestinal vagal afferent firing rate in vitro, selective knockout of GLP‐1R receptors in the nodose ganglia increased meal size^84^ and a subdiaphragmatic vagotomy abolished the inhibitory effect of PYY and GLP‐1 on food intake.^85^

Many of these gut peptides are also produced in and/or have receptors in the brain and can also impact the hedonic system. For example, intra‐cerebral infusion of CCK decreased food intake.^86^ Further, injection of CCK into the NAc reduced VTA intracranial self‐administration of a rewarding electrical stimulation suggesting CCK attenuates VTA‐derived reward signaling.^87^ Subsequent studies suggest CCK1‐Rs are involved in the development of conditioned reward,^88^ whereas CCK2 receptors are involved in food reward responses.^89^ GLP‐1 and its receptors are also expressed in central feeding centers, such as the hypothalamus,^90^ hindbrain matrix,^91^ and lateral parabrachial nucleus (LPBN) of the posterior nucleus,^92^ directly impacting appetite. However, GLP‐1R is also expressed in multiple hedonic areas of the brain,^93^ such as the mesolimbic system (e.g., VTA, NAc, PVT and lateral spectrum [LS]), impacting on the motivation to consume food and conditioned reward responses. Delivery of the GLP‐1 agonist exendin‐4 into the NAc core decreased operant responding for sucrose,^94^ via suppression of dopamine signaling^95^ and thus a reduction in the reinforcing properties of the reward pathway. Further, activation of GLP‐1R in the PVT reduced food intake, motivation and food seeking^96^ and activation of GLP‐1 in the BNST reduced food intake in the dark phase.^97^ In addition, activation of GLP‐1R in the LS reduced food intake in ad libitum fed mice and reduced operant responding for sucrose in fasted mice.^98^

##### Gastric

Ghrelin is a multifunctional hormone involved in glucose and lipid metabolism as well as, in the context of this review, its ability to stimulate food intake.^99^ Primarily, ghrelin is produced by endocrine cells (X/A‐like cells) in the stomach.^100^ It is secreted before a meal^101^ and during fasting,^102^, ^103^ then inhibited once food has been consumed.^101^ Ghrelin stimulates food intake through both peripheral and central mechanisms, which has been reviewed previously.^99^, ^100^ Briefly, in the periphery, ghrelin has been shown to decrease the sensitivity of gastric vagal afferents in female mice,^104^ which could enable reduced perception of fullness and increased food intake after a fast. Centrally, ghrelin acts within the ARC where it stimulates NPY/AgRP neurons,^105^, ^106^, ^107^, ^108^ signaling to the PVN to increase food intake. Conversely, ghrelin also indirectly inhibits POMC via GABA inputs from the NPY/AgRP neurons.^105^, ^107^

Ghrelin also impacts on multiple hedonic pathways to influence food intake.^93^ For instance, ghrelin engages with the mesolimbic dopaminergic pathway, with administration of ghrelin into the VTA or NAc increasing dopamine‐mediated food intake^109^ through increased motivation to work for food^93^ and stimulates food intake by influencing the LHA, where it indirectly stimulates orexin neurons through regulation of neuronal projections from the ventral subtemporal regions of the hippocampus.^108^ Further, ghrelin had an orexigenic effect in rats fed ad libitum but reduced food intake in fasted rats^110^ when administered into the amygdala. In addition, administration of ghrelin into the lateral parabrachial nucleus (LPBN) increased intake of a standard chow diet but not lard or sucrose^111^ suggesting the impact of ghrelin on the LPBN is directed toward consummative rather than appetite behaviors.

Brain imaging studies have identified how gut hormones affect brain regions involved in regulating appetite. For example, in a human fMRI, the infusion of ghrelin has been shown to alter BOLD responses to food images in the amygdala, OFC, anterior insula, and striatum. These areas are known for their role in assessing the reward value of food‐related signals. Additionally, the effect of ghrelin on the amygdala and OFC has been linked to the self‐reported hunger levels of participants. These findings suggest that ghrelin's metabolic signals might promote eating by enhancing the hedonic and motivational responses to cues associated with food.^25^ This relationship between appetite‐regulating areas and ghrelin was confirmed in a recent systematic review of 10 fMRI studies, where ghrelin was positively correlated with activation in the pre‐frontal cortex (PFC), amygdala and insula and with increased hunger feelings. Whereas subcortical areas like the thalamus, hippocampus, striatum, and hypothalamus were negatively correlated with ghrelin levels.^112^ A more recent systematic review conducted only on individuals of normal weight, confirmed these regions, and highlighted the OFC as a significant brain area modulated by ghrelin.^113^

#### 2.2.4 Adiposity‐related endocrine signals

Although not technically part of the gut–brain axis, hormones secreted by adipose tissue also impact food intake regulation. For example, the satiety hormone leptin is secreted by adipocytes, with circulating levels positively correlated to body fat mass.^114^ As such, leptin is key in energy metabolism whereby higher concentrations of leptin in the plasma signals decreased food intake and reduced resting energy expenditure.^115^, ^116^ In lean mice and mice deficient in leptin (ob/ob mice), leptin treatment reduces calorie intake.^117^, ^118^, ^119^ Similarly, in humans deficient in leptin, leptin therapy reduces food intake^120^ and further in lean healthy subjects, leptin administration prevents the increase in food intake following refeeding after a fast.^121^ These homeostatic effects are mediated centrally, with leptin receptors localized in regions including the ARC, PVN, VHN, and LHA and outside the hypothalamus including the NTS and midbrain.^116^ To date, there is substantial knowledge on the leptin signal transduction pathways within these regions.^114^, ^122^ Briefly, food intake is altered through leptin effects on the long form of leptin receptor to activate the Janus‐activated kinase pathway.^122^ This leads to inhibition of NPY/AgRP neurons, through the recruitment of histone deacetylases and a decrease in AgRP expression, and activation of POMC/CART neurons, through the recruitment of histone acetylases and an increase in POMC.^123^, ^124^

There are also leptin receptors in the hedonic system, where leptin has a suppressive action on food‐induced reward.^125^, ^126^, ^127^ For example, activation of leptin receptors in the NAc decreased dopamine release, while silencing of leptin receptors increased dopamine release, sucrose preference and food intake.^125^, ^126^ Further, activation of leptin receptors within the VTA decreased food intake.^125^

### 2.3 Advances in gut–brain axis research: Pharmacological studies

Studies on the gut–brain axis have led to significant advances in incretin‐based weight loss drug therapy. For example, dipeptidyl peptidase 4 (DPP‐4) resistant synthetic analogues of the naturally occurring GLP‐1 hormone have gained significant attention in recent years for their role in weight management and obesity treatment.^128^, ^129^ These analogues enhance insulin secretion and inhibit glucagon release, thereby improving glycemic control, beneficial for individuals with type 2 diabetes.^130^ Several neuroimaging studies have explored how GLP‐1 receptor agonists influence the brain activity associated with appetite and satiety. For example, Farr et al. used fMRI to examine how GLP‐1 receptor agonists modulate brain reward circuits in response to food stimuli in overweight and obese individuals, finding a reduction in the activation of brain regions involved in food reward.^131^ This modulation likely contributes to their efficacy in reducing appetite and aiding weight loss.^131^ Similarly, Kulve et al. assessed the effects of endogenous GLP‐1 and a GLP‐1 analogue on brain responses to food consumption in obese individuals, finding that both forms of GLP‐1 influenced neural networks related to the reward and sensory perception of food, suggesting a mechanism by which GLP‐1 analogues may aid in weight loss.^132^ Further, GLP‐1 analogues impact brain areas associated with food cravings, indicating their role in diminishing the appeal of high‐calorie foods.^133^

While GLP‐1 analogues have demonstrated promising results from animal models to clinical studies, translating the findings of CCK1 receptor agonists in animal models to clinical application has been more challenging.^134^ Significant hurdles have been linked to the stability of CCK1 agonists and their side‐effects,^135^ with research and development ongoing to enhance the stability and tolerability of these compounds.^136^

Due to the robust orexigenic effect of ghrelin, several studies investigated a potential therapeutic use of this peptide as a potential treatment for appetite stimulation (e.g., in anorexia) and gastric motility. As highlighted earlier, fMRI studies have revealed that ghrelin administration increases activity in brain regions associated with reward and food intake.^25^, ^112^, ^113^ However, while these imaging studies suggest that ghrelin modulators do affect brain circuits related to eating behavior, their role in clinical settings may be more complex and less effective than initially thought.

Other studies have investigated the effects of combined and separate infusions of satiety gut hormones. For example, dual incretin therapies have been gaining increasing attention for promoting weight loss, with dual receptor agonists (e.g., tirzepatide), targeting both GIP (glucose‐inhibitory polypeptide) and GLP‐1 receptors, outperforming GLP‐1 receptor agonists alone in terms of weight loss and improving glycemic control in patients with type 2 diabetes.^137^ In addition, combinations of CCK peptides with GLP‐1 receptor agonists have shown significant potential in rodents for the treatment of both type 2 diabetes and obesity.^138^ Acute co‐administration of PYY and GLP in obese human subjects was shown to elicit at least an additive effect on food intake at tolerated doses of the drugs in combination.^139^ Thus, combination therapies for the treatment of obesity hold promise for weight loss interventions in clinical practice.

Collectively, these studies highlight the importance of neuroimaging in understanding the mechanism of action of gut peptides in suppressing appetite and promoting weight loss. They also validate effectiveness, dosing, and treatment protocols for weight loss and glycemic control. Neuroimaging can further aid in understanding individual variability in response to incretin‐based treatments. By observing how different brain patterns correlate with the effectiveness of gut peptides, personalized treatment plans can be developed for more effective weight management. However, these studies do not rule out possible peripheral actions of gut peptides on reducing food intake, such as through activation of gastrointestinal vagal afferents.^128^ Understanding the mechanisms of action of gut peptides in controlling appetite and aiding weight loss remains crucial in advancing targeted drugs strategies with fewer side effects toward clinical application.

### 2.4 Nomenclature of targets and ligands

Key protein targets and ligands in this article are hyperlinked to corresponding entries in http://www.guidetopharmacology.org, the common portal for data from the IUPHAR/BPS Guide to PHARMACOLOGY,^140^ and are permanently archived in the Concise Guide to PHARMACOLOGY 2019/20.^141^, ^142^, ^143^, ^144^

## 3 CONCLUSION

Recent advancements in neuroimaging techniques allowed the investigation of the complex network of brain regions responsible for regulating eating behavior. This network encompasses homeostatic, reward, and executive control regions, which interact with signals related to energy balance, external environmental factors, and an individual's genetic makeup. However, to create successful weight management strategies, it is imperative to conduct further research that delves into understanding how overweight, obesity, and weight loss impact the central nervous system regions responsible for regulating eating behavior, as well as the underlying biological factors influencing these changes.

## AUTHOR CONTRIBUTIONS

G.S.C., A.J.P., and S.E. all contributed to the writing and editing of the manuscript.

## ACKNOWLEDGMENTS

None.

## CONFLICT OF INTEREST STATEMENT

The authors declare no conflict of interest.

## ETHICS STATEMENT

The information discussed within this article has been conducted in accordance with ethical guidelines.

## Data Availability

Data sharing is not applicable to this article as no new data were created or analyzed in this review.
